# Mining of primary raw materials as the critical foundation of ‘sustainable’ metals: a wicked problem for technology innovation clusters

**DOI:** 10.1098/rsta.2023.0241

**Published:** 2024-11-04

**Authors:** K. R. Moore, E. Marquis, K. Shanks, F. Wall

**Affiliations:** ^1^ Camborne School of Mines, University of Exeter, Penryn Campus, Treliver Road, Penryn, Cornwall TR10 9FE, UK; ^2^ Environment and Sustainability Institute, University of Exeter, Penryn Campus, Treliver Road, Penryn, Cornwall TR10 9FE, UK

**Keywords:** responsible mining, supply chains, circular economy, natural capital

## Abstract

A transition to a more sustainable human–nature system is inextricably linked to raw materials production, if economic growth is to be maintained or increased by the emergence of new, energy- and metal-hungry technology innovation clusters. The dependence on mined raw materials is a wicked problem for societies vulnerable to negative ecological impacts and for global power bases wanting to secure access to an increasing array of feedstocks. We interrogate the issue of what constitutes a sustainable metal from a triple perspective: (i) the characteristics of ore deposits and the primary extractive operations that supply critical raw materials; (ii) the impediments for complex and interacting supply chains to maintain critical (and other) metals in use; and (iii) the lack of transparency in supply chains that makes it challenging for customers to avoid resources that have been produced by unsustainable and poor practices. We examine existing and emerging structures for resource management to explain the limits to the circular economy and what constitutes a meaningful systemic structure for primary production by responsible mining. We call for the inclusion of a standardized statement of the ‘natural capital’ embodied in R&D for technological materials as a means to create transparency about what constitutes a sustainable metal.

This article is part of the discussion meeting issue ‘Sustainable metals: science and systems’.

## 1. Introduction

The cumulative sustainability of metals is significantly impacted by mining at the start of the supply chain. An individual mine for metal or metalloid is an inherently unsustainable venture since the ‘ore’ minerals of interest are extracted and are not returned to the exact site of extraction. The timeframe over which the industrial activity occurs is called ‘life-of-mine’, and it can be very short (even less than a year, depending on economic and other conditions) or several hundred years. Metallic minerals may remain at the site in the mine waste or ‘gangue’ if they are not profitable to extract at the time of mining or, in the case of historic mining, there was no use for companion minerals/metals. Moreover, processing of ore minerals does not recover 100% of the metals that it is profitable to extract. Sustainability is therefore linked to the efficiency of metal recovery by minerals processing, influencing whether excess waste is created and whether natural resources are squandered by poor practice. The impacts of poor recovery and mine waste deposition relate to the transfer and release of potentially toxic metals from subsurface environments into surface environments. The impacts can be realized at mine sites or at sites of enhanced processing that are distant from the site of extraction.

The term ‘sustainability’ in mining is applied to environmental and societal issues, which can be significant. Mining inevitably causes perturbation to the environment over some spatial scale and time interval; it can divide communities, and the withdrawal of the mining industry can undermine a dependent community. The life-of-mine can be shortened by negative environmental and societal impacts, and associated legal or physical tensions, that leave economic ore deposits unmined. As a consequence, environmental and societal sustainability issues are the primary risk [1] that is acknowledged by mining companies, and they dictate whether or not mining can proceed in many cases. The term ‘responsible’ mining is often used to describe best practices in mining operations that address sustainability issues.

This manuscript aims to highlight how the sustainability of metals that we use in modern technological societies hinges on responsible practice in the mining sector. We approach the issue using the critical raw materials that are used in increasing diversity and quantity to enable the energy transition and the associated technology cluster. In the first instance, we define what constitutes criticality, and we consider the geological and extractive challenges associated with criticality assessments of the mined feedstocks for alloys, batteries and permanent magnets, and metamaterials. Subsequently, we investigate strategies for raw materials management and the limits to maintaining critical raw materials in the circular economy that could be addressed with further research and changing attitudes. We consider the mechanisms by which greater awareness of mining can be fostered where metals are described as sustainable, given (i) the challenges of following supply chains back to responsible (or otherwise) mining; and (ii) the increasing proximity of mining operations to communities as regions seek to implement their critical minerals or critical raw materials strategies.

## 2. The criticality of raw materials

The manuscripts in this volume place a strong emphasis on decarbonization, through sustainable design and utilization of renewable energy, for the production of sustainable metals. Renewable energy provision is linked to mining in the energy–minerals nexus [2–4]: energy is needed for raw materials production, as well as manufacturing, but the infrastructure and technologies for the low carbon transition cannot be manufactured without recourse to raw materials production. Production volumes of some energy-technology metals have changed markedly over the last decade: Li production has expanded by 208%, while lead and natural graphite production have declined [5]. New supplies by mining are coming online, but they are not projected to keep pace with the fourfold to sixfold increase in metal demand for the green transition, and raw material shortages are anticipated [6].

The energy-technology metals are important for innovation in materials and energy engineering because they have specialist properties and high functionality to create the necessary components. They are described as having high economic importance (EI, table 1) to modern manufacturing in consumer regions. The Critical Raw Materials (CRMs) are determined to have significant supply risk (SR, table 1) as well as economic importance. The EU, US, Japan and the UK all have CRM risk assessments and strategies to secure access to raw materials [4,8,49–52], which vary as a function of trade agreements and regional ore deposit availability. In the EU, criticality risks in the strategic renewables, e-mobility, defence and space sectors have been analysed by considering the CRMs that comprise batteries, fuel cells, wind turbines, traction motors, photovoltaic cells, robotics, drones, three-dimensional printing and ICT [53]. Large manufacturing companies in multiple sectors, including automotive and food sectors [54], have strategic working groups to manage their material flows. Designations of criticality therefore vary according to geographical region or nation, or the material needs of an industrial sector or individual company [8,50,53]. They allow for the formulation of strategies by decision-makers to secure supply.

**Table 1.** Selected examples of CRM and SRM, as cited in the text, and a summary of their use and geological sources. Overall criticality index CI_CRM_ is from [7]; NA, data not available. Supply Risk (SR) and Economic Importance (EI) are quoted from [8]. LSM, large-scale mining. ASM, artisanal and small-scale mining.

CRM	SR	EI	CI_CRM_	example use	geological source	references
Al / bauxite	1.2	5.8	NA	important to lightweight the (automotive and aerospace) transport fleet. Solar PV and other energy applications	bauxite is the ore for alumina and Al. It forms by residual enrichment of the least mobile components of weathering alumina-silicate rocks, most effectively in tropical and subtropical climates	[9,10]
Sb	1.8	5.4	3.59	alloys with lead (batteries, low friction metals, metal sheathing, etc), fire retardants, pigments, GST-bearing metamaterials	mostly hydrothermal^a^ deposits, associated with magmatic and metamorphic processes; they can be associated with gold deposits. They are often discontinuous in extent and not commonly reaching ore grades. Production concentrated in China	[11–13]
As	1.9	2.9	NA	used as a doping agent (GaAs) in semi-conductors, it is critical due to economic importance. Also used in bronzing, pyrotechnics, hardening shot, glass manufacture, wood preservatives. Use as pesticide is controlled due to its toxicity	largely recovered by roasting of arsenic minerals in polymetallic hydrothermal and Cu–Au and Au ores (various countries), and also by hydrometallurgy from Co–Ni–Au–As hydrothermal vein-type ore (Morrocco). Also recovered from smelter flue dust	[8,14,15]
Be	1.8	5.4	3.26	used in metallic alloys, as pure metal and in ceramics. Applications are mostly in the aerospace, electrical and electronic sectors. Additional sectors are mechanical, oil and gas drilling/exploration	Be-hosting minerals occur in trace amounts in sub-alkaline granitic pegmatites^a^ and alkaline igneous rocks. Recent LSM commercial mining has been concentrated in the USA, Kazakhstan and China. There is online ASM production associated with gemstone mining	[16]
Bi	1.9	5.7	4.01	low-temperature solders, free-machining alloys, triggering devices for sprinkler systems	wide range of deposit types, but global production of Bi is concentrated at the Núi Pháo mine (skarn^a^ deposit) in Vietnam and as a by-product of Pb and W mining in China	[17]
Ce La Nd (LREE)	4.0 3.5 4.5	4.9 2.9 7.2	4.36 4.25 NA	Nd in permanent magnets in wind turbines, information and communication technologies. Small volume uses of LREE in ceramics, glass production and phosphors	magmatic ores hosted in, and laterites^a^ developed above, carbonatites^a^ and alkaline rocks; hydrothermal veins, IOCG^a^ and metamorphic ores Concentration of production from the only ‘giant’ ore deposit of REE (Bayan Obo in China) though they are geologically available globally	[18–20]
Co	2.8	6.8	3.22	high-performance metal alloys; components in LiBs; digital storage devices; hydrogen production; semiconductors; desulphurization catalyst; paints and coatings; machinery and plastics	by-product of Cu or Ni production from: stratiform^a^ sediment-hosted Cu–Co deposits; magmatic Ni–Cu–Co sulphides; high grade metasedimentary rocks, including Ni–Co laterites. Central African copper belt is an important global source region by LSM and ASM. Rare mining of Co as primary product (Morocco)	[21,22]
Cu	0.1	4.0	NA	electronic components and infrastructure in all RE generation; catalyst; numerous alloys (e.g. brass and bronze) with malleability, ductility and resistance to corrosion. It is a SRM due to low substitutability	global distributions of varied geological occurrence. Porphyry^a^ Cu deposits are high tonnage but low grade for LSM. Notable exceptions are IOCG^a^ (Olympic Dam, Australia) and carbonatite-hosted (Palabora mine, RSA). Small, high grade hydrothermal ore deposits are currently subeconomic to mine	[8,9,23]
Dy Er Sm Tb	5.6 5.6 3.5 4.9	7.8 3.5 7.7 6.4	NA NA NA NA	added to permanent magnets to extent their working range (see LREE under Ce). Er also used in metamaterials	the occurrence can be similar to the LREE Ce, La, Nd but with a tendency to concentrate more in alkaline rocks than carbonatites, and in ion-absorption deposits in weathered granites	[20,24–26]
Fluorspar (and F)	1.1	3.8	1.75	HF acid; flux in steel-making and electrolytic reduction of alumina to Al; opaque enamels and glass; electronic uses (semi-conductors); catalysts in petrochemical production; pharmaceuticals, herbicides, pesticides; processing of nuclear fuel. HCFCs and PTFEs with environmental and health implications	fluorspar is the commercial name for the mineral fluorite CaF_2_, found in many geological environments. Economically, it is sourced from: hydrothermal veins, in association with Pb, Zn, Ag and barytes; metasomatic replacement^a^ ore deposits; weathered enrichment zones above vein ores; residual granites and pegmatites, alkaline magmatic rocks and carbonatites	[8,27]
Ga	3.9	3.7	3.327	eutectic alloys with low temperature applications (e.g. thermometers); permanent magnet manufacture; arsenide and antimonide semi-conducting compounds with military, wireless communications, PV and battery, LED applications; pharmaceutical; petrochemical catalysts	a by-product primarily of the alumina-refining stage in the processing of bauxite (the ore of Al, see above), but also low recovery from processing of sphalerite (the ore of zinc). The greatest Ga enrichment in bauxite is associated with the greatest weathering, particularly of the alkaline igneous rocks or associated with iron-rich horizons	[28]
Ge	1.8	3.6	3.48	fibre-optics, infrared optics; catalysts for colourless PET; various electronics/solar applications (e.g. metamaterials for PV and thermo-PV cells, integrated circuits, detectors); medical (e.g. chemotherapy); metallurgical (e.g. alloys with Sn and Ag)	rarely concentrated to economic grade, as a by-product of Cu, Zn and Pb production. It is concentrated with host metals most widely in sulphide (less commonly in oxide) ores: e.g. volcanogenic massive sulphide^a^, porphyry copper and hydrothermal vein deposits. It can also be concentrated in coal deposits	[29]
Li	1.9	3.9	NA	lithium-ion batteries (LiBs) for medium- and large-scale energy storage; alloyed with Al for lightweight construction; fluxing agent in production of enamel and ceramics; chemical applications in, e.g. lubricants, pharmaceuticals	supply dominantly by pegmatite-sourced lithium hydroxide and salar deposits. Lithium hosted in (volcano-) sedimentary clay deposits, granitic micas and directly from groundwater have yet to be proven at industrial scale, globally	[30,31]
Mg	4.1	7.4	4.08	alloys for castings and wrought or formed materials, with widely varying applications from packaging and structural, to transportation and military. As a powder, it is used for desulphurization of iron and steel. Mg metal is a reducing agent in Ti, Zr, U and Be production	calcined dolomite is the dominant source. Alternatives are magnesite, magnesium silicate minerals such as olivine and serpentine, and brines that have undesirable contaminants. The dominant process to separate magnesium is currently the silico-thermic method, due to availability of low cost ferro-silicate (as a reductant) in China	[32]
natural graphite (C)	1.8	3.4	3.38	natural graphite accounts for 80% of the battery-grade requirement. In addition, it is used in refractories, for steel-making, foundry facings, brake linings, lubricants and pencils	vein, flake or microcrystalline graphite, with flake graphite being most mined. Metamorphism and reduction of carbonate minerals. Subsequent mobilization and migration leads to hydrothermal vein deposits. Less than half of graphite is produced by mining, with the remainder synthesized from coke	[33–35]
Ni	0.5	5.7	NA	nickel-based high-performance alloys in chemical and aerospace industries, for heat exchangers and missile components; Ni pig iron produced from low-grade laterite ores, for stainless steel; Cu–Ni–Fe–Mn alloys for coins; plating and many catalyst uses; batteries, e.g. lithium nickel manganese cobalt oxide (NMC)	magmatic Ni–Cu–PGE deposits that formed by crystallization at high-temperature in magma chambers and ancient lava flows supply most of global demand. Laterites are expected to become an increasingly important source as demand increases and processing challenges are resolved. Smaller tonnage and grade ores are metasomatic replacement and hydrothermal vein type	[36,37]
Nb	4.4	6.5	3.60	low-alloy steels; Nb–Ti and Nb–Sn wires in superconducting magnetic coils; lithium niobate coatings on glass computer screens; Nb carbide cutting tools	ore deposits are globally widely dispersed, but production is from carbonatites concentrated (approximately 90%) in Brazil, with most of the remainder from Canada. They also occur in alkaline to peralkaline granites and syenites, and LCT pegmatites, coupled to Ta	[38,39]
Pd Pt Rh Ru (also Ir and Os)	1.5 2.13 2.4 2.8	8.1 6.9 8.6 5.5	3.06 3.05 3.68 3.48	platinum group elements (PGE) are auto-catalysts, chemical process catalysts, including hydrogen fuel cells; jewellery, electronics and specialist medical alloys	igneous processes in high-temperature magmas concentrate all the PGE. Supply concentred in South Africa (frequently associated with chromite deposits), Russia, USA and Zimbabwe. Pt and Pd are concentrated in sufficient abundances within porphyry systems for economic recovery from Cu refinery anode slimes	[40]
Sc	2,4	3.7	4.36	enormous potential as lightweight alloys for transportation; scandium iodide used in Hg vapour lamps; radioactive Sc as a tracer in oil refining, and to detect leaks	limited by-product of conventional U and Ni extraction processes; mining as primary commodity planned from laterite^a^ deposit in Australia	[41]
Ta	1.3	4.8	2.76	electronics (capacitors) for portable electronic devices; in cutting tools, high temperature alloys and corrosion-resistant equipment in the chemicals industry	columbotantalite (coltan) in granite related, e.g. LCT (Li- Cs-Ta) pegmatite deposits, Rwanda and eastern DRC, Australia, where Ta > Nb. The largest Ta deposits occur in pegmatite swarms	[38,39]
Te	0.3	3.8	NA	alloys with Cu, stainless steel and Pb. Used to vulcanize rubber, to tint glass and ceramics, as a catalyst in oil refining, in rewritable CDs and DVDs. Thin film CdTe photovoltaics are the market driver	majority as a by-product of electrolytic Cu refining, largely in China, with less by-production from Zn ores. Three Au-(Ag-)Te mines (two in China, one in Sweden) are minor producers of Te. A super-large independent Te deposit is reported in China, of debated geological origin	[42–44]
Sn	0.9	4.5	NA	protective coating for steel containers; Pb- or Zn-alloys; solders for joining pipes and circuits; glass-making; PV technology and lithium-ion batteries; superconducting magnets; liquid-crystal displays; toothpaste and dental medicine; non-toxic coatings for wood; stabilisers for polyvinyl chlorides (PVCs)	majority from tin ore provinces in larger granitic belts: primarily from magmatic-hydrothermal vein deposits, associated with late-stage fractionated tin granites, pegmatites and tin porphyries. Smaller production is from placer^a^ deposits arising from erosion of the primary ores. Indonesia, China and Myanmar dominate production but tin-granite districts are globally available	[45,46]
W	1.2	8.7	3.47	alloys for wear-resistant coatings; high strength carbide and steel alloys; mill products (wire, sheet and rods); light-bulb filaments, electrodes, vacuum tubes, heating element; chemical applications including pigments, catalysts, fireproofing of textiles	associated with granite intrusions or medium- to high-grade metamorphic rocks. Multiple processes of formation of primary ore deposits create a wide variety of forms including veins and stockworks,^a^ disseminated greisen,^a^ skarn, porphyry deposits. Some secondary placer deposits have been worked. Although deposits are well distributed globally, China has the largest resources and reserves	[47]
V	2.3	3.9	2.17	alloying agent for Fe and steel (>80%) for automotive, construction and nuclear industries; Ti alloy additive; catalyst in sulphuric acid production; battery applications; oxides as pigments for ceramics and glass, and producing super-conducting magnets	globally distributed main deposits are: vanadiferous titanomagnetite deposits in magmatic layered intrusions; sandstone-hosted U-V deposits; shale-hosted V deposits; base metal-related vanadate deposits. Particularly in oxidized supergene^a^ areas. However, placer and surficial U-V type mineralization might become important	[48]

^a^ See glossary of geological terms.

**Figure 1.**
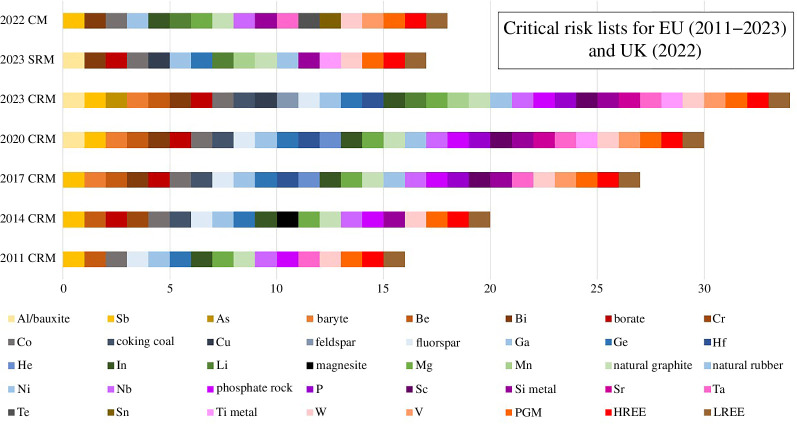
Critical risk lists for the EU (CRM: Critical Raw Materials 2011–2023) and UK (CM: Critical Minerals 2022). The 17 strategic raw materials (SRM) for the EU are a subset of 34 CRM and comprise the materials expected to grow exponentially in terms of supply, which have complex production requirements and thus face a higher risk of supply issues. HREE and LREE are presented here separately for all surveys, but they are cited as total REE in the 2022 CM and 2011 CRM lists. PGMs are presented as one group here, but the 2022 CM list includes Pt and Pd as separate commodities. 2020 CRM includes bauxite only, but this was subsequently changed to be inclusive of Al/bauxite. Sources of data are [8,50].

Figure 1 shows how the number and range of commodities designated as CRMs by the EU has increased from 14 to 34 in 12 years [8]. The British Geological Survey has identified 18 minerals and metals as having high criticality for the UK economy in the first survey of criticality outside of the EU [50]. In the first EU risk list in 2011, the CRMs were metals, semi-metals and the mineral fluorite. The addition of coking coal (metallurgical coal for steel manufacture), phosphate rock and natural rubber in 2014 and 2017 lists extended the CRM portfolio into bulk rocks and biogenic materials. The addition of bauxite (the ore for aluminium) and copper in 2020 and 2023 extended the portfolio of critical and strategic raw materials (SRMs) into those bulk metals used in large quantities, described by the World Bank as the cross-cutting metals used across the technologies of the low carbon transition [9]. The increase in number of CRMs is largely due to an increase in the scope of the EU assessment to a wider range of commodities [8] and greater recognition of a reliance on imports for the increasing material demands of the green energy transition. Just as increasing knowledge has expanded the range of materials considered at critical risk of short supply, so too have commodities been removed from the risk list. Indium is recovered from zinc smelters and it was a CRM of the EU from 2011 to 2020, and in the UK in 2022. The criticality of In in the EU was reduced in the 2023 assessment by a change of supporting data from solely domestic supply to a combination of global and domestic supplies, more precise allocation of In in the high-tech sector and domestic production meeting or exceeding the current demand [55].

The risks to imports are largely upstream and extractive (i.e. related to mining) or processing bottlenecks in supply chains that are exacerbated by global tensions (e.g. trade negotiations or military conflict), which has informed strategy. The UK Critical Minerals Strategy [56] aims to grow UK domestic production capabilities, collaborate with international partners and enhance international markets to make them more responsive, transparent and responsible. The EU Critical Raw Materials Act [57] has a similar ethos, and aims for domestic capacities along the supply chains by 2030, whereby annual EU consumption should feature more than 10% domestic extraction, more than 40% domestic processing and more than 15% domestic recycling activities. In addition, not more than 65% of the Union’s annual consumption of each strategic raw material at any stage of processing should be from a single third country. The UK and EU strategies will act to diversify the geographical spread of raw materials production, which requires that raw materials production is closely scrutinized. Moreover, the EU conflict minerals regulations (applying to Sn, W, Ta, Au [58]) sets a standard that consumption in an end-manufacturing society has responsibility, by driving the supply chain, to avoid supporting negative consequences of raw material production in producer regions. Extension of these ideas to the topic of sustainable metals, for systems and science, demands investigation of the raw materials requirements for materials engineering and innovation.

## 3. Upstream CRM requirements for materials engineering feedstocks

If the sustainability of metals that we use in modern technological societies hinges on responsible practice in the mining sector, then it is important to recognize the use of mined feedstocks in modern innovation. To understand the geological underpinning of innovation in materials engineering, we can take as examples some areas where criticality of metals is inherent, such as traditional or innovative alloying, battery and permanent magnet compounds, and innovation for composite materials and metamaterials (table 1).

### (a) Metal alloys

In the first instance, it is possible to choose one alloy over another using an assessment of the alloy criticality index (CI_A_) [7]:


(3.1) $${\text{CI}}_{\text{A}}=\sum_{i=1}^{n}{\text{CI}}_{{\text{CRM}}_{i}}\frac{\text{wt}{\%}_{{\text{CRM}}_{i}}}{100},$$

where *n* is the number of CRMs in the alloy chemical composition, CI _CRM ‘i’_ is the overall criticality index and wt% _CRM ‘i’_ is the proportion of the CRM ‘i' in the alloy, measured in weight percent. The methodology was successfully applied in the cases of Be-, Al-, Mg- and Ti-alloys and steels, for bicycle forks and con-rods in high-performance engines [7]. More significantly, the replacement alloys may have lower alloy criticality index but may yet include a specific CRM that becomes subject to supply shortage. The criticality of raw materials can fluctuate widely over relatively short timescales, so the choices made in one year may increase risks the following year, and the use of the criticality index needs to be dynamic.

Dynamic changes in criticality and the validity of substitutions are illustrated by the potential for substitution between critical metals Ta, Nb and V, which are all used in steel alloys. Ta has the lowest supply risk (SR = 1.3) in 2023, and it is a conflict metal because profits from some mines are used to finance conflict, primarily in the Democratic Republic of the Congo (DRC). Due to rising prices, it was substituted out of some applications in the early 2000s [38]. With excellent strength and creep performance at high temperature, good conductivity and chemical resistance, approx. 90% of Nb production is used to make alloys for conventional and some bespoke applications including superconductors and nuclear fuel rod cladding [38,59]. However, the substitution of Ta by Nb caused alloys to fail military specifications in many important end uses [38], and there is a trade-off with supply risk, since Nb has higher SR (4.4 in 2023, table 1) being produced dominantly by one mine at Araxá in Brazil. A black swan event that impacted the mine in Brazil might be mitigated by use of global stockpiles but would be unlikely mitigated by production at any other mine sites, since the time taken to start mining operations is incompatible with a timescale of demand–supply imbalances [60]. Substitution of Nb by V (SR = 2.3 in 2023) is possible as a rapid response, with steels maintaining function at similar temperatures for mainstream uses. However, *V* criticality arises from more than 70% of raw material co-production mainly from the slag of iron ore largely in China and Russia, such that supply chains could rapidly be disrupted by changing economic and political relations. Thus, decision-making for metal substitutions using equation (3.1), based on metrics that may change very rapidly, requires that there is some underpinning knowledge that will allow for a rapid reappraisal of criticality in the light of geopolitical changes that can outpace intermittent, metric-generating assessments.

### (b) Batteries and rare earth permanent magnets

With transitioning energy systems, the enabling battery (and magnet) metals are experiencing heightened demand with Ni, Co, (Nd) and Li increasing by 40%, 70%, (130%) and 200%, respectively, from 2017 to 2022 [6]. These metals are included in the SRM (figure 1) subset of CRMs. The use of lithium (table 1) in rechargeable lithium-ion batteries (LiBs) increased from 0% in 1991 to 80% in 2007 [61]. Li is favoured due to its high charge-to-density ratio, which enables its wide use in portable electronics and electric vehicles. Li content remains fairly stable in a variety of LiB types but other components vary dramatically in concentration between LiBs. Due to price volatility and supply concerns, Co has been reduced and Ni increased in concentration in Ni–Mn–Co (NMC) LiBs, and LiMn_2_O_4_ (LMO) and Li–Fe–P (LFP) LiBs have Co-free chemistry [36]. With shifting chemistries, new criticalities can emerge. For example, fluorophosphate-based chemistries [62], such as LFP, are reliant on fluorine predominantly sourced from fluorspar (95%; [63]), which is considered critical by the EU and the USA. As alternatives to LiBs, Na-based battery compositions have been developed that do not have the same charge-to-power ratio but can be highly suitable for city-based shorter-range EVs.

There are large uncertainties in the future demand for lithium and a variety of criticality scenarios that include undersupply crises [30,64–71]. Pathways to make Li ‘uncritical’ in the long term [30] include ramping up hard-rock (pegmatite-hosted) mining for Li, diversifying resource types and/or the increase in consumption of Li such that it becomes a bulk metal produced by large, globally dispersed operations [30,67,69]. There are assumptions built into the scaling up of production and consumption beyond decision-making about winning technologies (and their composition). The large overheads of multinational mining corporations usually mean that technology metals with uncertain demand scenarios in a small-scale market are not an attractive business proposition. Few large mining companies have developed interests in Li production: Rio Tinto’s Jadar project had permits and licences revoked by the Serbian government in 2022 due to societal opposition; Imerys has plans to begin production at Imerys British Lithium (Cornwall) by the end of the decade, and at the Beauvoir site in central France by 2028. Investment in Li mining [72] is extremely volatile and drops when supply pressures ease, so the dominant small and medium enterprises are challenged to scale up operations.

Rare earth element containing magnets, such as Nd–Fe–B and Sm–Co, are favoured for energy-generation and transfer purposes such as turbines, portable electronics and EVs. With the need to decrease dependencies on REE, some motor manufacturers have switched to alternative REE-free, induction-based motors, so that there is some uncertainty in projected industry-specific future demand. Production of Nd, Dy and Tb from primary sources can lead to oversupply of other REE, such as La and Ce. Promoting demand for elements in oversupply, by development of high-volume and high-value applications (e.g. alloys), could reduce waste and improve supply chain stability of all REE by potentially diversifying their economics [18]. In this case, new stocks of REE are becoming available as a consequence of technological substitution. For example, fluorescent lights containing Tb have been superseded by LEDs. The Tb that is recovered by hydrometallurgy is used to replace primary (mined and refined) Tb and Dy in permanent magnets [73]. Now there are efforts underway by multinational companies to produce REE: e.g. Rio Tinto producing REE-bearing monazite from its ilmenite mineral sands project at QIT Madagascar Minerals.

### (c) Composite alloys: metamaterials

**Figure 2.**
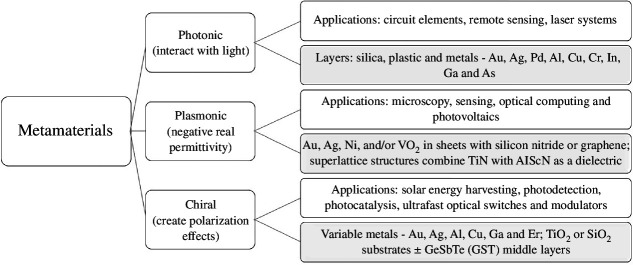
Scheme showing three dominant groups of metamaterial, their properties and applications [74,76], and their component parts (shaded boxes).

Metamaterials are three-dimensional or two-dimensional (metasurface) structures composed of at least two different materials with a response or function that is not possible to achieve with any individual constituent material. Metamaterials replicate mineral or crystalline lattices, being based on the replication of a unit cell with sub-wavelength dimensions that result in a variety of functional properties including: electromagnetic, acoustic, magnetic, mechanical/structural, thermal or chemical [74]. However, many metamaterials combine metallic and dielectric (e.g. plastic) components in layers, coatings and chiral arrangements to create new material properties. Viable products based on metamaterials are being rapidly developed and commercialized [75], with specialist applications that demand the use of metallic critical raw materials with specialist properties [74,76], though in low quantities relative to alloying and batteries. Examples of metamaterial types, their applications and component commodities are illustrated in figure 2. This is by no means an exhaustive list of the elements incorporated into metamaterials or consumed during their manufacturing; this is a currently expanding field and here we focus on a snapshot example of the technologies’ potential impact on material criticality. There is an opportunity in directing these currently rapidly developing technologies towards the use of non-critical elements where possible [76].

The precious metals are important but their production is not critical because commodity price remains high through economic downturns and production remains steady. The high commodity price means that gold mining attracts multiple stakeholder and varies from informal, low-technology artisanal mining from alluvial deposits through industrial small- and medium-scale mining to state-of-the-art large-scale hard rock mines. The commodities used in metamaterial fabrication (the shaded boxes of figure 2) that are subject to supply concerns fall into three dominant groups, based on their production origins:

1. Critical and Strategic bulk metals (included in SRMs, figure 1), e.g. Al and Cu. The cross-cutting metals of the energy transition [9] are needed in already vast and yet rapidly increasing quantities, which demands global-distributed operations in large tonnage ore deposits, with a consistent feed into processing plants. The volume of materials that are extracted, even at low unit cost of production, make it an attractive proposition to multinational mining companies with large overheads.
2. Critical technology metals by mining (CRMs and CMs, figure 1), e.g. Pd, Cr, Ni, As, V, Sb, Co. Where the large-scale mining paradigm is applied to CRMs, global demand is satisfied by a few mines. This results in commodity-specific mining monopolies and geographical bottlenecks of extraction and processing. The economics of smaller mines are unattractive to multinational corporations and there are a larger proportion of junior companies involved in extraction.
3. Critical technology co-product and by-product metals: REE, Ga, Ge, Se, Te, In, Co, Pd. The REE are co-produced and must be separated, resulting in overproduction of the REE that have low demand relative to their proportion in the ore. Where by-product critical raw materials (e.g. Ge, Bi and Sc) are largely produced during refining or smelting of bulk metals such as Zn, Cu, Pb, Al and Sn, then the economic drivers of the carrier metal dictate whether by-production can be increased [77]. See Steinlechner (this volume) for a more in-depth analysis of by-product metals.

Several metals can be produced either as primary or by-product commodities, e.g. Bi [17], Co [21] and PGEs [40]. Additional and/or alternative capacity is being planned by the opening of new mines where the CRMs are the primary commodity of interest, rather than ramping up production purely as by-products (e.g. for Sc, table 1).

### (d) Geometallurgy and mining of ore deposits

Materials engineering for the functionality of end-user technologies and requirements for consumer products to be affordable indirectly drive requirements for mining innovation, which ensures that the unit cost of production of raw materials remains low. This means that commodity prices do not necessarily reflect the true value of natural capital in economic discourses, which has long been recognized [78]. Nevertheless, the mechanisms to ensure that raw materials are affordable at the start of the supply chain include a consistent feed into continuous and highly efficient crushing, grinding and processing operations. Consequently, although the raw materials are geologically available, not all geological mineral deposits are economically viable. The ultimate goal of geological exploration has been to find the largest mineral deposits that will be most lucrative and will be classified as ‘world-class’ ore deposits.

For bulk metal SRMs that have been mined for centuries, the availability of the largest tonnage and highest-grade ores is dwindling. For the CRMs required in smaller quantities, the world-class ore deposits will geographically concentrate global production. Now that critical mineral strategies and Acts [56,57] in consumer regions require a proportion of raw material supply by domestic extraction, alternative geological deposits that are unlikely to satisfy the ‘world-class’ criteria are under investigation [19,22]. This may be a development for greater sustainability, since more rock is crushed and processed in low-grade ores than in high-grade ores, at high energy consumption and high waste production. A movement to smaller ore deposits at higher grade, with more selective extraction of material, may contribute to decarbonization of mining practices and reduction in mine waste generation.

**Figure 3.**
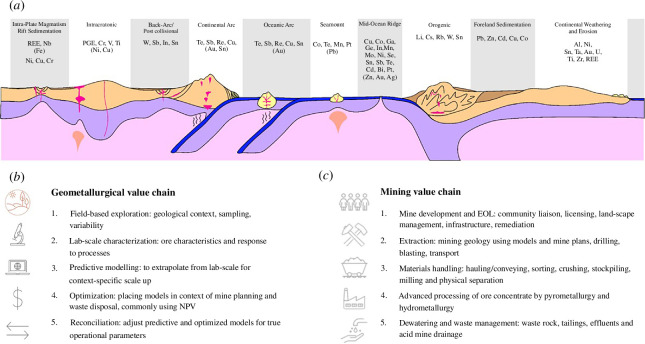
Diversity of environments of ore deposit formation (*a*) requires that geometallurgical (*b*) and mining (*c*) value chains adapt to geological, mineralogical and metallurgical variability. The value chains are not static linear chains and require ongoing validation and adjustment throughout the life-of-mine. Planning for dewatering and waste management, and end of life (EOL) of mine should, in best practice, inform all stages of mine development.

Economic ore deposits are very diverse in nature and occur in many tectonic environments (figure 3*a*), such that the geometallurgical value chain (figure 3*b*) starts with field investigations. Geometallurgy combines geological and geostatistical information with extractive metallurgy testwork in order to provide predictive modelling for minerals processing plants where, together with further downstream processing (including smelting) in the supply chain, the extraction value of metal and semi-metal by-products accrues. The ease with which the ore minerals are liberated from rock depends on the ore mineral type, its textural relations to gangue minerals in the mineral assemblage and the grain size of materials. Exploration proceeds by selectively focusing on the dominant minerals or metals of interest, in order to prove the financial viability of an ore deposit to investors. This means that CRMs are not always adequately factored into sampling and assaying programmes, and thereby will be excluded from economic assessments. One of many examples of a CRM that is often omitted from surveys is Bi [17], which has substitutability of no more than 10% in metallurgical and other applications, recycling rates of less than 1% and for which the EU and the USA are 100% reliant on imports of refined metal [17].

**Figure 4.**
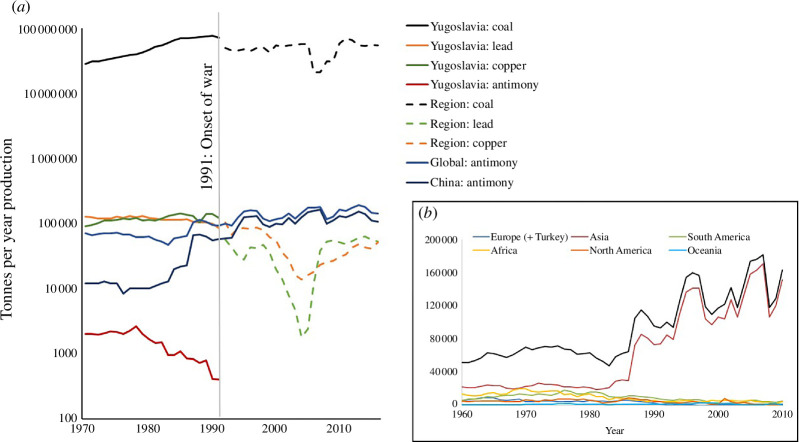
Raw materials production of selected commodities prior to, and following the onset of the Yugoslav Wars in 1991 (*a*) and global production of antimony from 1960 to 2010 (*b*). Data derived from Mineral Statistics database, constructed and maintained by the British Geological Survey. Note that the logarithmic scale reduces the apparent perturbations of the commodities used in higher volumes, which were significant.

Investor concerns about ESG at mine sites, to minimize risks to mine development, coincide with conducive corporate structures and can have the impact of driving investigations (research, monitoring and enforcement) into responsible practice at mine sites. The reality is that the mining value chain (figure 3*c*) at the mine site of a responsible producer will factor community engagement and co-creation of appropriate solutions between mine operator and community, throughout the mine cycle. Investment can have a positive impact on economy, with positive repercussions for society and environment. An example is raw material production in the Balkans, which suffered a black swan event with the onset of the Yugoslav Wars (figure 4*a*). Coal, Cu and Pb production continued after the initial shock, then subsequently deteriorated further with the loss of market and infrastructural challenges, and started to improve after the onset of foreign direct investment and humanitarian aid for reconstruction efforts. By contrast, Sb production from vein deposits ceased altogether, despite its significance as a CRM used in alloys, battery manufacture and metamaterials. A steadily decreasing production of Sb in the former Yugoslavia mirrored that of increasing production in China (figure 4*b*). The global geopolitical economic climate of CRM production was not amenable to the re-establishment of antimony production immediately after the wars, with dwindling production from Sb mines across Europe due to a competitive economic climate rather than due to exhaustion of ore deposits. Current initiatives to improve the security of supply of critical raw materials, patterns of global production relative to manufacturing bases, and market price mean that antimony is once again a commodity of interest for exploration and development in the Balkans.

**Figure 5.**
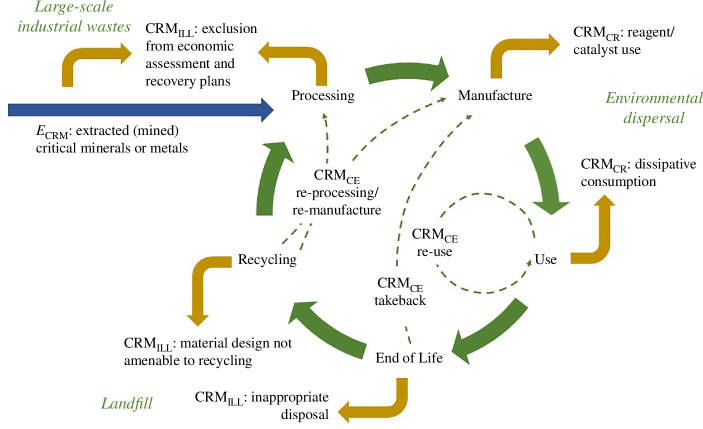
Schematic depiction of a simplified circular economy (CE) model, in which the extracted (mined) critical minerals or metals (*E*_CRM_) that enter the economy are divided between those that enter the circular economy (CRM_CE_) and those that are lost to the circular economy either by consumption practices (CRM_CR_) from which they cannot be recovered or by inefficiencies, losses and landfill (CRM_ILL_). Dominant positions of CRM_CR_ and CRM_ILL_ are shown, though they can occur throughout the CE. The dominant characteristics of waste and environmental fate are indicated.

The key messages from the case studies above are that (i) responsive and dynamic material substitution can alter, but does not eliminate, criticality; (ii) investment volatility and risk management affect whether scale-up of production can happen, by small-, medium- or large-scale enterprises; (iii) ore deposits are being explored and developed to extend production capacity to satisfy demand for a sustainable supply of metals; and (iv) a sustainable supply of metals for materials engineering is impacted by international relations, strong national mining policies, and civil and/or military conflict.

## 4. Systemic structures for raw materials management

Responsible production of raw material is not restricted to the mining sector at the start of the supply chain. Early raw materials management systems were proposed on a whole systems basis, particularly in relation to supply chains and wastes. The reduce–reuse–recycle slogan entered modern parlance in the mid-1970s, and it was embedded in the urban mining concept, which ‘provides a systematic management of anthropogenic resources stocks and waste (products and buildings), in the view of long-term environmental protection, resource conservation, and economic benefits’ [79]. The urban mine is further described in terms of material losses in the system using a mass balance equation [79]:


(4.1) $$\Sigma d_{i}=E-\Delta R-\Delta L-l,$$

where Σ*d*_i_ is the diffuse mass emissions/loss that should be controlled, *E* is the primary extracted raw material that should be minimized, Δ*R* is the recycled and reused material, Δ*L* is the recovered material from landfill mining and *I* is the immobilized material in final sinks and geological repositories. The urban mining concept is not synonymous with the umbrella circular economy concept [80], which includes strategies to maintain products in use for a longer time interval (by reuse and repair) and to foster growth and opportunities by using more secondary materials in production cycles. Urban mining is simply the process of reclaiming compounds and elements from any kind of anthropogenic stocks including mine wastes [81], which is important because it extends the urban mine from population centres into rural environments, and it extends the potential sources of CRMs from active mine sites into legacy ore deposits. There are centuries-worth of mine wastes where polymetallic ore deposits were mined for bulk metals and CRM-bearing minerals were discarded in waste, because they did not have the commercial applications that have proliferated in the technology cluster associated with the low carbon transition.

In practical terms, the concept of the circular economy is applied to discretized CRM supply chains and industries such as the battery metals initiatives [82–85] that strive to vertically integrate stakeholders to ‘close the loop’ and recirculate materials into the supply chain. The strategy is appropriate since the increasing demands are for a specific combination of technology metals, subject to the definition of technology design [30]. However, recycled metals that enter the economy via the minor metals market can leave the discretized circular economy through alternative uses. Examples of uses that can consume or diffuse CRMs into the environment [86] include pharmaceuticals and cosmetics (such as Ba, Li), pigments and chemical dyes (such as Cr, Ni), fertilizers (such as P), catalysts and reagents (such as Cr, REE, Pt and other PGM). So the circular economy can reduce the volume of raw material that needs to be extracted, but the extent to which this occurs depends on the diversion of recycled material to consuming and diffusing uses (figure 5), as described by the mass balance:


(4.2) $$E_{CRM}-CRM_{CE}=CRM_{CR}+CRM_{ILL},$$

where *E*_CRM_ is extracted (mined) critical minerals or metals, CRM_CE_ are the critical minerals that enter the circular economy, CRM_CR_ are critical minerals that are consumed resources that become unavailable to the circular economy and might be dispersed to the environment, and CRM_ILL_ are the critical minerals that are lost due to inefficiencies, losses and landfill. Opportunities to increase environmental and economic resilience can therefore be fostered by identifying where CRM_CR_ and CRM_ILL_ can both be reduced. Criticality risks along the supply chains in a whole systems view can be premised on identifying the critical metals that can be described in circular economy framings and those that are sequestered and disposed. In other words Σ*d*_i_ [79], as the diffuse mass emissions/loss that should controlled, can alternatively be viewed in terms of the end-use applications of CRMs.

Intervention points to increase input of *E*_CRM_ (figure 5) in the circular economy are volumetrically largest at single raw materials production sites. In the first instance, a research focus on the ore deposits that are neither ‘world-class’ nor requiring the development of untested new extractive or processing routes are most likely to secure regional supplies in the near future, since it is time-consuming to demonstrate fit-to-purpose and environmental compatibility of new and innovative techniques. In the second instance, a shift in geometallurgical value chains to enhance multi-commodity exploration and processing streams for co-production of both primary and legacy ore deposits might have the potential to contribute significantly to security of supply.

Intervention points to increase retention of CRM in the circular economy include the end-use applications of CRMs that consume resources (CM_CR_) and recycling of complex materials to reduce CRM_ILL_ (figure 5). The efficiency of applications that constitute resource consumption (CM_CR,_ figure 5) or dispersive use (Σ*d*_i_) could be researched to examine whether it is possible to reduce material use. Alternatively, the space exists for improvement mechanisms for recovery from remediated effluents, from flocculants that remove toxic chemicals from wastewaters, by bioremediation using tolerant, metal-absorbing plants or lo-tech clean-up solutions where chemicals are absorbed into sawdust [87,88]. CRM_ILL_ (figure 5) demands attention because the recycling rates for CRM are so much lower than for bulk metals [77,86,89]. Poor recovery arises from domestic hoarding and incineration, the bonded nature of materials (e.g. metamaterials or batteries) and small throughputs, and processing costs of recycling related to energy barriers (e.g. photovoltaic cells) [90,91].

For legacy deposits (Δ*L*), and mixed shredded streams from recycling plants and/or landfill (Δ*R*), material characterization is the key to understanding secondary resources across the urban mine [92]. Material characterization is foundational to the well-established concept of geometallurgy that, in this sense, acts as a way of thinking that bridges all aspects of primary and secondary supply chains. It is thus a systemic structure that is also a practical way of thinking with clear meaning [92]. It demonstrates that the geological, mineralogical, metallic and compound properties cannot be decoupled from the processes of resource utilization across the supply chains, nor can they be decoupled from the cumulative environmental impacts of consumption. The production-consumption duality is an important thinking structure that challenges the perception that economic growth and consumption can be decoupled from the environmental impacts of resource production [93], and it challenges the notion that metals can be produced sustainably [94,95].

## 5. Transparency and sustainability in raw materials management systems

**Figure 6.**
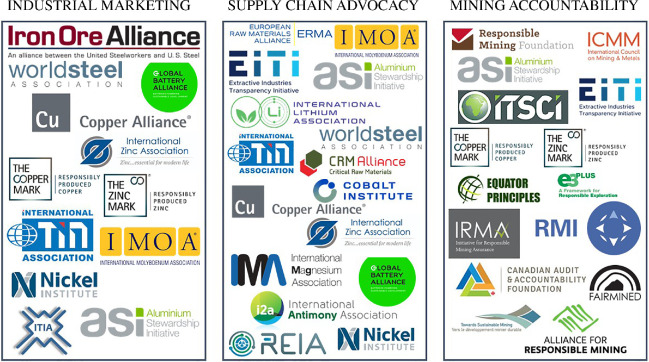
Representative selection of stakeholders in the value chain of mined commodities, which are either proactive in the marketing of extractive companies, advocate for unified approaches along the supply chains, or act (either independently or within supply chain advocacy groups) to audit mines and ensure industrial accountability.

If management of any primary or secondary CRM resource production cannot duplicate past ways of operating on a purely economic basis, then the joined-up thinking to reframe ideas and approaches [94,96], and intervention points, requires a flow of reliable information. It is challenging to trace raw materials through the supply chain but efforts are underway to design the methodologies for the creation of Digital Product Passports for CRMs, and thereby understand the circular value of systems and materials, and to improve recycling [95]. The proposed Digital Product Passport is focused at the manufacturer level for information exchanges and to trace the materials in components, to design for disassembly and to ensure product performance [95]. If the aim of materials passports is to increase circularity and thereby sustainability, the credibility of the sustainability credentials of the systems-wide approach is once again dependent on the sustainability criteria of the original input feedstocks, and whether they have been produced responsibly. Gathering of data about the feedstock inputs faces two significant challenges relating to the reliability of data about mined raw material inputs. The first challenge is the diversity of materials in modern technological products and the concomitant diversity of oversight bodies that supply information (figure 6). The second challenge is the lack of transparency from intermediate product and component manufacturers, where this might be due to either commercial sensitivity and marketing based on performance rather than composition, or a lack of will to reveal supply chains that stem from extractive operations that have poor responsibility credentials [97].

Stock exchanges have minimum standards for public reporting of progress on exploration and mining projects by their listed companies. Perhaps the best-known example of these CRIRSCO codes are the Australian Joint Ore Reserves Committee (JORC) code and the Canadian stock exchange National Instrument 43-101. The inclusion of ESG criteria in these CRIRSCO codes varies. The Pan European Reporting Code (PERC) was updated recently to include more ESG information and likewise JORC is also updating assessments of an ore reserve using Environmental, Social and Corporate Governance (ESG) criteria. Good ESG performance therefore enables companies to demonstrate their sustainability credentials to investors. It is simultaneously a decision-making tool for investors wanting to make conscientious investment choices in responsible companies, or investors simply seeking to minimize their investment risks. How well the measures cited in ESG statements are implemented in real terms depends on the company management and culture as well as regulation, licencing, monitoring and enforcement in the mining jurisdiction. A company that addresses only one aspect of sustainability is considered to demonstrate ‘weak sustainability’, while companies that responsibly implement practices and policies that address all impacts have ‘strong sustainability’ [98]. The responsible mining index [97] is an independent measure of how responsibly mining companies perform and it reveals that there are wide differences.

Figure 6 shows a representative selection of bodies that either promote extractive industries, strive to bridge supply chains or to ensure responsible production, spanning the breadth of mining practices from large-scale mining to artisanal and small-scale mining (e.g. Fairmined). Since most technologies require multiple components, each with multiple feedstocks/commodities, there are many organizations through which components and materials must be traced in order to understand the cumulative mining impacts of products. Full transparency is therefore difficult to achieve. Note that some of the assurance schemes act across multiple supply chains: the Copper Mark, for example, also assures responsible practices across the copper, molybdenum, nickel and zinc value chains. Some of the stakeholders appear in multiple clusters in figure 6, for example the Aluminium Stewardship Initiative, which holds mining companies to account and certifies companies across the entire value chain, if they meet and maintain the required standards that they promote. Companies can subsequently use their certification as a marketing tool to show their credibility.

In an effort to harmonize reporting on natural resource projects, the United Nations Economic Commission for Europe’s Expert Group on Raw Materials (EGRM) developed the United Nations Framework Classification (UNFC). Resource projects are classified based on environmental-socio-economic viability (E-category), technical feasibility and field project status (F-category), and geological confidence (G-category). Building on the UNFC, the ERGM subsequently developed the United Nations Resource Management System (UNRMS). The UNRMS provides a voluntary framework for integrated resource management (UNFC), uniformly applicable to all forms of natural capital. Rather than fragmented decision-making for independent projects or sectors, the UNRMS emphasizes multi-stakeholder environmental-socio-economic viability in whole resource-based areas, countries or regions.

**Figure 7.**
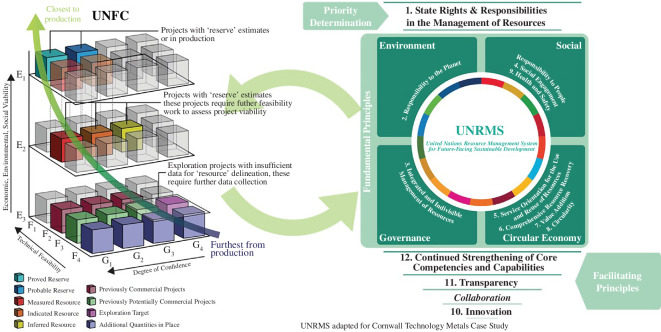
Interplay between the United Nations Framework Classification (UNFC) and United Nations Resource Managment System (UNRMS). The UNFC acts to harmonize reporting of resources and reserves for a wide range of materials types, whereas the UNRMS (after [99]) builds on these data to guide the management of resources for sustainable development; enactment of such a system aims to strengthen the development of resources in a manner that is consistent with ‘strong sustainability’.

The UNRMS is designed as a toolkit for the sustainable management of resources. UNRMS is framed around 12 principles for good resource governance (figure 7) that can be applied at various levels (project, regional and national). By including environmental, social and governance criteria as well as circular economy principles and transparency, the UNRMS strives towards strong sustainability [98] that is inclusive and acts beyond the ESG criteria for investment.

Application of the UNRMS to critical metals projects in Cornwall, SW England, highlighted the complexity of sustainable resource management, but was deemed a useful tool to identify areas that require focus or improvement to ensure sustainable development at regional level. These included aspects such as individual project environmental impact assessments; implementation of strategic environmental assessments to ascertain the impact of industrial ecosystems; and that, to develop a sustainable sector, there may be short-term costs to ensure the appropriate skills, infrastructure and technologies are available, which will provide benefit on a regional basis [99]. However, the UNRMS is still in the early stages of use, with further case studies planned to investigate its application in different geological, political and jurisdictional contexts (e.g. Zambian Copperbelt): these studies will further assess the UNRMS’s effectiveness to guide sustainable development related to natural and anthropogenic resources.

## 6. The limits to consumption: a wicked problem

The three driving factors that will act to spread mining more widely and in closer proximity to population centres with different mindsets about extraction are (i) exponentially increasing supply of more diverse types of ore deposit; (ii) strategies for increasing domestic production and securing supplies from more broadly dispersed production centres; and (iii) limits to, and inefficiencies in, the circular economy to offset primary raw materials production. Gardiner *et al.* [30] assert that the increase in production will drive materials away from criticality, i.e. by scaling up production of technology metals until they become bulk metals. They state an assumption that ‘techno-economic challenges and even environmental, social and governance (ESG) challenges can be overcome, and if not, the demand for the given metal is structurally adjusted by substitution, thrifting, or other correctives’. There is further in-built assumption that the challenges about primary production are correctly identified and, if they cannot be addressed, that relevant knowledge is effectively translated along supply chains so that the appropriate corrective measures are implemented. The wicked problem of whether or not innovative engineering based on consumption of resources will outpace whole-system capacity to extract (by open-cast and underground mining) sufficient materials in an environmentally and socially tenable manner is not one that is sufficiently recognized.

The mindset that technological development will solve any or all issues in a crisis is either optimistic or exclusive, whereby in the latter case, the concerns of local impacted communities are marginalized in the development of ‘green sacrifice zones’ [100]. The term describes the decision-making in power bases to dedicate lands for extraction, against the wishes of inhabitants, for the benefit of consumers. It amounts to a form of green neocolonialism that exacerbates intranational inequalities [101]. Furthermore, it perpetuates the Resource Curse [102], which describes the inability of regions to retain mineral wealth and thereby foster development: the exportation regionally or internationally of low-cost, largely unprocessed, raw materials fails to improve the livelihoods and well-being of producer communities since the value is added to the raw materials further downstream in the value chain. The UNRMS (figure 7) provides a mechanism by which to try and counter some of these challenges, grounded in the context of regional knowledge and understanding of environment, micropolitical and cultural context. However, it cannot be relied upon to resolve challenges in the absence of strategies to reduce material dependencies. In this context, the term ‘sustainable metal’ might recognize that there is short-term high impact during extraction, but long-term ‘value’ in the economy, if raw materials are managed/used appropriately as valuable natural capital and not lost to dispersion, waste and unaccountability.

If companies use ESG and certification schemes to attract investors and customers, investors use ESG as a decision-making tool for project selection, and strategists use criticality assessments as enabling information to protect economies, the question arises whether sustainability agendas are being used to propagate ‘business as usual’ and amount to little more than greenwashing. Tayebi-Khorami *et al.* [103] highlight the social responsibility of mining companies, but they align societal aspirations with circular economy aspirations and thereby frame waste management as a means to reduce company liability. Harvey [104] warns against blurring appropriate boundaries between firms, governments and communities because it will risk poorer outcomes than focusing on in-reach, whereby companies focus on behavioural change at whole organizational level in order to secure trust. Fundamentally, trust also requires transparency and communication and this is problematic when all mining and processing practitioners do not implement consistently responsible practices [97]. Trust and the reliability of information further becomes problematic when a large number of stakeholders are involved along the supply chain (figure 6), though it is fostered at local level by the UNRMS (figure 7).

The wider value chain of innovation clusters significantly includes research and development, which can be siloed and does not necessarily proceed with raw materials requirements in mind. For example, material substitution to manage risks for industrial practitioners can shift demand between critical raw materials and create unintended consequences that, if properly recognized, require regular short-term adjustments in order for businesses to remain competitive. In this case, criticality can be fostered through demand fluctuation and price volatility and risk is maintained or increased for the end-users who cannot engage with material substitutions for functional reasons. The wicked problem is perpetuated where the natural capital underpinnings of the supply chains and the socio-environmental impacts of mining are not widely enough recognized and communicated in the innovation clusters. We do not intend that innovators should be challenged to discover the origins of the raw materials they use in R&D, or be involved in developing digital passports. However, a simple standardized statement of the ‘natural capital’ embodied in R&D for technological materials would serve as a means to raise awareness of geological/mineral dependency. The Raw Materials Information System (RMIS) [105] is a suitable resource for the task because it provides simple and accessible, updated metrics describing the production characteristics of the raw materials, CRMs and other raw materials such as iron and steel, from a European perspective.

There is a greater propensity for innovative activity to spatially cluster in the early stages of the industrial life cycle and to disperse during the mature stages with congestion effects [106]. The congestion effects in the context of innovation for the low carbon transition may be strongly linked to escalating demand for critical raw materials, and the risks of short supply from producer nations to manufacturing and consumer nations have a high probability of increasing. The recognition of risks to security in global innovation clusters informs organization change and international management strategies for scientific and technical development, by marketing innovation and industrial, economic and social policies [107]. With this backdrop, the innovators operating in academic research environments can play a pivotal role in the Helix Models of open innovation [108]: adoption of a standardized statement of ‘natural capital’ usage by researchers working with industry and government would serve to foster dialogues about what constitutes a ‘sustainable metal’, to articulate the geological/environmental ‘load’ of innovation and serve as a trigger for wider discourses about the future of consumption.

## 7. Conclusions

This manuscript interrogated the issue of what constitutes a sustainable metal from a critical raw materials perspective. We investigated the systems that have variously been employed to manage raw materials, to limit the negative impacts of extraction and consumption, to improve transparency in raw materials supply chains, and the mechanisms needed to move forwards from greenwashing to meaningful transformation for sustainable systems. This is important because all mines are not equal, in terms of production and processing, and not all mining practitioners adhere equally to responsible best practices. It is far from certain that environmental and societal tensions will be adequately resolved to ensure sufficient supplies of geological ‘natural capital’ to accommodate escalating material demands for a sustainable low-carbon transition.

The issues around transparency and tracing of metals along supply chains that we have described suggest that no metal can be described as ‘sustainable’ if the primary mined origins are not adequately accounted. The circular economies for alloys, batteries and permanent magnets, and metamaterials are limited by complex interactions and complex bonding of components that is essential for functionality; by the ability to close loops and vertically integrate value chains; and by the diverse losses from the circular economy caused by dispersive consumption. The strategies to secure CRM supplies are created by consumer nations, but the geographical widening of production centres might increase societal rejection of mining, simultaneously with expectations for increasing consumption. Engineering for reduced raw materials consumption is more immediately accessible than changing behaviours for reduced consumption in society, which has the potential to impact economic growth and thereby national security.

## 8. Glossary of geological terms relevant to CRM occurrence

  — Carbonatite—a carbonate-rich igneous rock (crystallized from magmas) that is highly prospective for multiple CRMs, most notably Nb and REE but also phosphate, fluorite, zirconia and Cu.
  — Greisen—a quartz- and mica-rich rock that has formed where granite or pegmatite have been highly altered by the percolation of hot fluids that have a magmatic derivation.
  — Hydrothermal—a description of mineral veins that crystallized from ‘hot waters’ that flowed through rocks, with the veins described as ‘lodes’ where they filled space along fractures and faults, as ‘stringer lodes’ where many small veinlets surround major veins and the rock can be mined as a unit, or ‘sheeted veins’ where the filled fractures are parallel and separated by barren rock. See also greisens, metasomatism and stockwork.
  — IOCG—a family of iron oxide-copper-gold ore deposits that have a variety of geological and tectonic settings.
  — Laterite—a type of soil that forms from rocks by weathering, which removes soluble rock components and concentrates less soluble ore minerals including those hosting CRMs (e.g. Al, Ni).
  — Metasomatism—the pervasive process of changing a rock through the introduction or removal of chemical components, usually by the action of fluid flow through a rock.
  — Pegmatite—a very coarse-grained intrusive igneous rock that forms in the later stages of crystallization of a (often granitic) magma chamber where the residual magmas are concentrated in elements that are incompatible with many common mineral structures, including Li, Cs and Ta (LCT pegmatites) and Nb, Y and F (NYF pegmatites).
  — Placer—an accumulation of ore minerals (e.g. native Au, Ti-minerals such as ilmenite, or REE-hosting monazite) that have physically eroded from source rocks and subsequently been concentrated by gravity separation from less dense gangue minerals in natural, usually alluvial or marine, settings.
  — Porphyry deposit—a large body of granitic to dioritic rock in which coarse-grained crystals are set in a finer-grained matrix and that has been fractured and invaded by boiling, metal-laden (commonly Cu) magmatic fluids.
  — Skarn—a metamorphic (solid state recrystallization due to high pressure and/or temperature) alteration or replacement adjacent to igneous rocks by hydrothermal waters that can transport and concentrate CRMs.
  — Stockwork—a complex system or network of randomly oriented and closely spaced veins so that it can be mined as one unit; common in, and associated with, many types of ore deposit, including porphyry Cu, greisen and VMS deposits.
  — Stratiform—a mineral deposit formed parallel to the bedding planes of a rock, though it does not have to be constrained to a single unit (strata bound) or the same age (syngenetic) as the host rock.
  — Supergene—zones of leaching and enrichment in an ore body, which occurs due to the preferential solubilities of metals in downwards percolating and oxidizing (meteoric) waters; will concentrate Cu at the water table, leaving Fe-rich residual laterites.
  — Volcanogenic massive sulphide (VMS) deposits—predominantly stratiform or mound deposits above stockworks, which form by exhalative (hydrothermal) processes associated with underwater volcanism, observed in real time as black smokers. They are rich in Cu and Zn with the potential CRM co- or by-products Co, Sn, Se, Mn, Te, Ga and Ge.

## Data Availability

The data used in the drafting of this manuscript are open access. Reports of the European Commission, the British Geological Survey, United Nations and OECD are available on their respective websites. The Mineral Statistics Database is supported by the British Geological Survey at https://www.bgs.ac.uk/mineralsuk/statistics/world-mineral-statistics/.
